# 

**Published:** 1862-03

**Authors:** 


### BOOKS RECEIVED.

*Commentaries on the Surgery of the War in Portugal, Spain, France, and the Netherlands, from the battle of Rolica in* 1808, *to that of Waterloo, in* 1815; *with additions relating to those in the Crimea in* 1854, 1855, *showing the improvements made during and since that period in the great art and science of Surgery on all the subjects to which they relate. Revised to October,* 1855, *by* G. J. GUTHRIE, F. R. S. *Sixth edition. Philadelphia:* J. B. LIPPINCOTT & CO., 1862. *For sale in Buffalo by* BREED, BUTLER & CO.—$1.50.

*Border Lines of Knowledge in some provinces of Medical Science; an introductory Lecture delivered before the Medical Class of Harvard University, Nov. 6th.* 1861, *by* OLIVER WENDELL HOLMES, M. D., *Parkman Professor of Anatomy and Physiology. Boston:* TICKNOR & FIELDS, 1862.

*Amputation of the Cervix Uteri, by* J. MARION SIMS, M. D., *Surgeon to the Womans' Hospital, New York.*

*Clinical Lectures on the Diseases of Women and Children, by* GUNNING S. BEDFORD, A. M., M. D., *Professor of Obstetrics, the Diseases of Women and Children, and Clinical Obstetrics, in the University of New York; Author of "The Principles and Practice of Obstetrics." New York:* WILLIAM WOOD, 1862.

---

### PAMPHLETS RECEIVED.

*Communications of the Rhode Island Medical Society for the year* 1861. *Published by the Society. Committee of Publication:* GEORGE L. COLLINS, M. D.

*Eleventh Annual Report of the Providence Reform School, for the year ending November* 30, 1861.

